# Impact of combining data from multiple instruments on performance of patient-based real-time quality control

**DOI:** 10.11613/BM.2021.020705

**Published:** 2021-04-15

**Authors:** Qianqian Zhou, Tze Ping Loh, Tony Badrick, Chun Yee Lim

**Affiliations:** 1 Engineering Cluster, Singapore Institute of Technology, Singapore, Singapore; 2 Department of Laboratory Medicine, National University Hospital, Singapore, Singapore; 3 Royal College of Pathologists of Australasia Quality Assurance Programs, Sydney, Australia

**Keywords:** quality control, laboratory management, moving median, moving average, average of normal

## Abstract

**Introduction:**

It is unclear what is the best strategy for applying patient-based real-time quality control (PBRTQC) algorithm in the presence of multiple instruments. This simulation study compared the error detection capability of applying PBRTQC algorithms for instruments individually and in combination using serum sodium as an example.

**Materials and methods:**

Four sets of random serum sodium measurements were generated with differing means and standard deviations to represent four simulated instruments. Moving median with winsorization was selected as the PBRTQC algorithm. The PBRTQC parameters (block size and control limits) were optimized and applied to the four simulated laboratory data sets individually and in combination.

**Results:**

When the PBRTQC algorithm were individually optimized and applied to the data of the individual simulated instruments, it was able to detect bias several folds faster than when they were combined. Similarly, the individually applied algorithms had perfect error detection rates across different magnitudes of bias, whereas the error detection rates of the algorithm applied on the combined data missed smaller biases. The performance of the individually applied PBRTQC algorithm performed more consistently among the simulated instruments compared to when the data were combined.

**Discussion:**

While combining data from different instruments can increase the data stream and hence, increase the speed of error detection, it may widen the control limits and compromising the probability of error detection. The presence of multiple instruments in the data stream may dilute the effect of the error when it only affects a selected instrument.

## Introduction

Patient-based real-time quality control (PBRTQC) is the laboratory quality control practice that monitors the performance of an analytical system through the use of patient results (*1*). It involves statistical manipulation of the live stream of patient results as they are generated from routine clinical care, *e.g.*, using data transformation, outlier treatment, and statistical algorithms, and comparing these data against pre-determined control limits (*1*, *2*). Compared to conventional quality control (QC), the main advantages of PBRTQC include having timelier (‘real-time QC’) detection of clinically important errors; reducing costs associated with conventional QC since it uses patient results already generated for routine clinical care; negating the issue of commutability. However, PBRTQC involves more complex statistics as well as software configuration. The use of PBRTQC is gaining increasing attention as an important tool in laboratory quality control repertoire (*1*).

Recently, there has been great activity on the optimization of various parameters for PBRTQC, its application in different laboratory settings, and the detection of different types of errors (*2*-*8*). At the same time, several reviews and recommendations have been published by the International Federation of Clinical Chemistry and Laboratory Medicine Working Group on PBRTQC to provide guidance in these areas to laboratory practitioners, and interested readers are encouraged to refer to them (*1*, *2*, *9*, *10*). However, the considerations for applying PBRTQC in multiple instrument scenario remains under-explored. This is particularly relevant for laboratories that employ multiple instruments for the measurement of the same analyte, or in scenarios where multiple instruments of the same type are deployed in the point-of-care setting, such as glucometer (*8*).

Using serum sodium as an example, this simulation study aimed to examine the error detection capability when running PBRTQC algorithms on a set of instruments individually and in combination.

## Materials and methods

**Figure 1:**
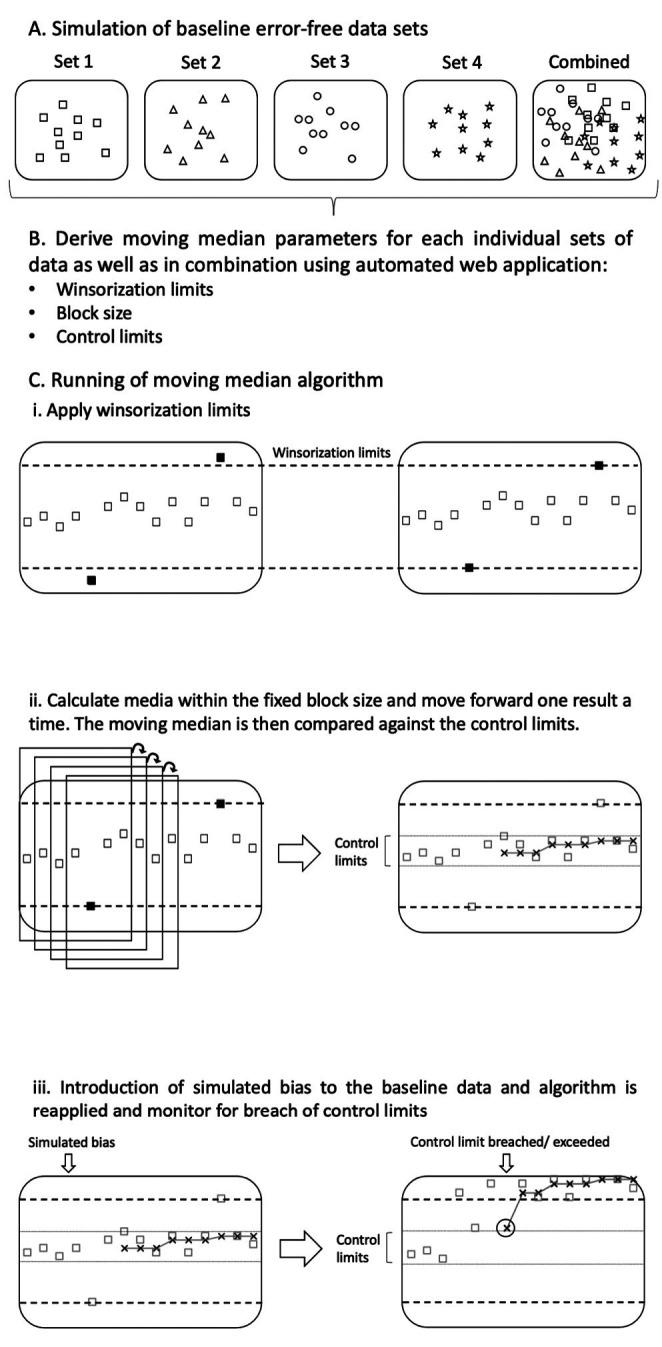
Workflow for this simulation study. In step A, four individual sets of baselines ‘error-free’ data as well as in combination are generated. In step B the moving median parameters are derived by feeding the baseline data into the web application. In step C i), the winsorization is applied to convert any outlier values into the corresponding predefined limits. In step C ii), the moving median algorithm is applied to the winsorized data using the block size and compared against the control limits. In step C iii), simulated bias is applied to the baseline data and the moving median algorithm is reapplied and monitored for breach of control limit (error detection capability).

This study only involves numerical simulation and is exempted from institutional ethic board review. An overview of the simulation process and PBRTQC algorithm of this study is provided in Figure 1.

### Simulation scenario

In this simulation study, four sets of 10,000 serum sodium measurements were randomly generated based on a Gaussian (normal) distribution with the mean and coefficient of variation (CV), expressed in % shown in Table 1. They represent the baseline (‘error-free’) patient data from four different instruments or ion-selective electrodes with a differing degrees of baseline population or inter-instrument / inter-electrode differences. Following this, analytical imprecision is introduced into each data point. The magnitude of the imprecision is drawn from a normal distribution with a mean of 0 and analytical imprecision (CVa) of 1.2%, and applied to the data points of all four instruments.

**Table 1:** Parameters used to simulate the baseline ‘error-free’ serum sodium data of the four sets of individual instruments and the optimized parameters applied on the moving median algorithm

**Scenario**	**Set 1**	**Set 2**	**Set 3**	**Set 4**	**Combined**
Population mean	140	140	137	139	139
Population standard deviation (SD)	4.3	3.5	4.3	4.8	4.4
Winsorization limit	132.3–147.5	133.8–146.2	129.4–144.4	130.5–147.4	131.0–146.6
Block size	150	115	80	150	150
Control limits	139.0–140.9	139.2–140.8	135.8–138.5	137.5–139.9	136.1–140.8
Annotated as set 1, set 2, set 3 and set 4. All values are in mmol/L.					

### Patient-based real time quality control algorithm and parameters

In this study, the moving median was selected as the PBRTQC algorithm as it was associated with a lower false positive rate and better error detection capability compared to the moving average for serum sodium (*2*). Under this algorithm, the median value of a fixed number of patient results (the block size) is calculated. The simulated data were introduced into a PBRTQC web application (https://pbrtqc.shinyapps.io/PBRTQCapp/) (*2*) to obtain the optimized block size and control limits based on an allowable analytical bias of 1.0% and false detection rate of 0%. The use of the web application ensures the automated, objective selection of the optimized PBRTQC parameters. As a new simulated result is generated, it is incorporated into the block while discarding the oldest result and the median value is recalculated. In this manner, the moving median is continuously calculated for the fixed block size with each new result. The moving median is then continuously compared against a pair of control limits with each new result available and when breached, will be flagged as error detected.

At the same time, winsorization is a statistical technique that seeks to reduce the effects of potential spurious outliers. This can be achieved by converting an outlier value to another (predefined) value. In this study, any value exceeding the winsorization limits will be converted to the values corresponding limits to minimize the effect of extreme values on the calculation of the moving median without sacrificing the data point (*2*). For example, if the upper winsorization limit is set as 150 mmol/L, a serum sodium result of 152 mmol/L will be converted into 150 mmol/L for the calculation of the moving median. The winsorization limits are set to 5% and 95% of the input data.

The optimized PBRTQC parameters are summarized in Table 1. To simplify the simulation, it is assumed that the instruments produced the patient results at the same rate. The four data sets are individually fed into the PBRTQC web application to obtain individually optimized parameters. The four data sets are also combined into a single dataset and then fed into the same web application to obtain a common parameter for the combined dataset.

### Bias simulation and performance measure

To assess the performance of the PBRTQC simulated analytical biases were iteratively added into the baseline ‘error-free’ patient data of the simulated instrument at a fixed interval of 100 results, as previously described (*2*, *8*). The bias was introduced from 1% to 20% in fixed increments of 1%. The performance was separately evaluated for each simulated instrument whether the PBRTQC algorithm was applied on the data of individual simulated instrument or combined. For the combined scenario, the bias was added into only one of the four simulated instruments. In total, 100 rounds of bias were introduced for each magnitude of increase.

The performance of the PBRTQC was evaluated using the median number of patients affected before error detection (MNPed), as previously described (*9*). Briefly, this performance matrix measures the median number of patient results (out of the 100 rounds of simulated bias introduction) between the point of bias introduction to the point at which the PBRTQC control limit was breached.

The probability of error detection was also derived by determining the number of times the PBRTQC control limit was breached after the introduction of bias over the times the bias was introduced (*9*).

The simulated instrument data is the sum of baseline (‘error-free’) value, imprecision, and bias. After the introduction of a small bias, it is highly probable that the total instrument error remains within total allowable error (TEa) 3% until the eventual error detection. On the other extreme, if the bias introduced is large, the moving average would breach the PBRTQC control limit within a small number of affected results, *i.e.*, the bias is detected quickly. The expected number of unacceptable final patient results with the total error which exceeds the TEa (*i.e.*, erroneous patient data), from the point of bias introduction to the point of bias detection, commonly termed as E(N_uf_) was quantified by the equation E(N_uf_) = total number of erroneous patient results / total number of bias introduced, where E(N_uf_) represented the expected number of erroneous patient results (*11*).

## Results

**Figure 2:**
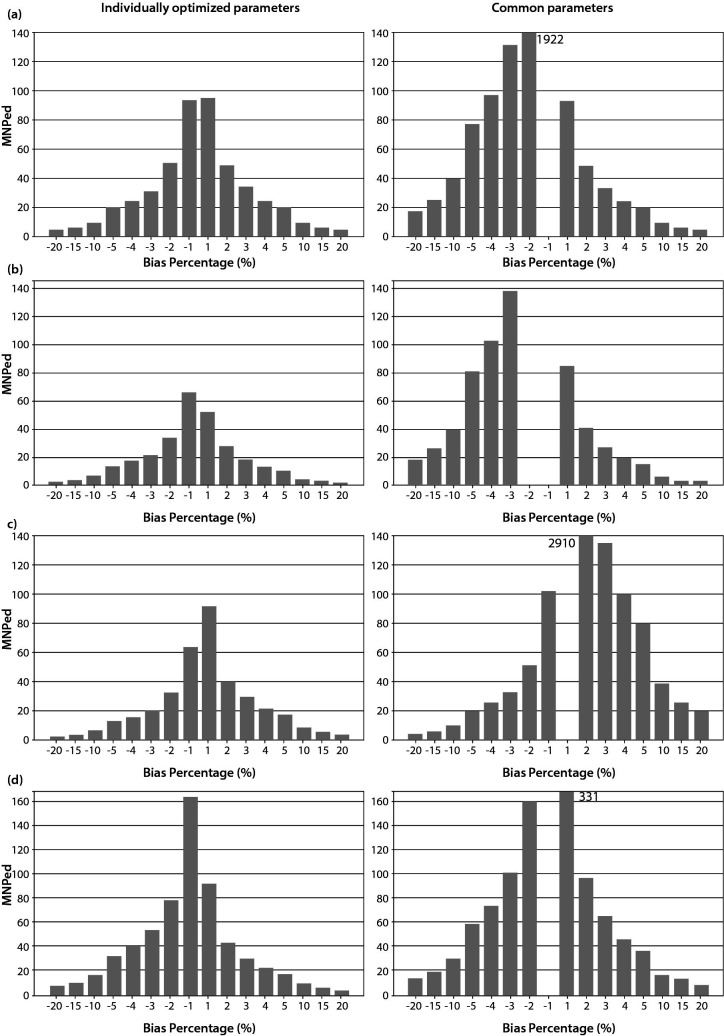
Median number of patient results before error detection with individually optimized and applied parameters and common parameters. On data: (a) set 1 (b) set 2 (c) set 3 (d) set 4. A higher MNPed indicates more patient results are affected by the bias before the error is detected. MNPed – median number of patients affected before error detection.

When the PBRTQC algorithm was applied to the combined patient results, it produced an unbalanced performance for the individual instruments. For instruments with a population mean closer to the control limit, they will detect positive biases with higher probability and earlier (lower) MNPed. The MNPed for a positive bias of 2% for instruments 1 and 2 is < 100 whereas the MNPed for instrument 3 is 2910 (Figure 2).

At the same time, the application of the PBRTQC algorithm on the combined patient results detected the simulated bias several folds later (higher MNPed) compared to when they were applied to the individual instruments. This can be seen in instrument 1, where a 2% negative bias was detected with an MNPed of 1922 when using combined patient results compared an MNPed of < 100 when the algorithm was applied to individual instruments (Figure 2).

**Figure 3:**
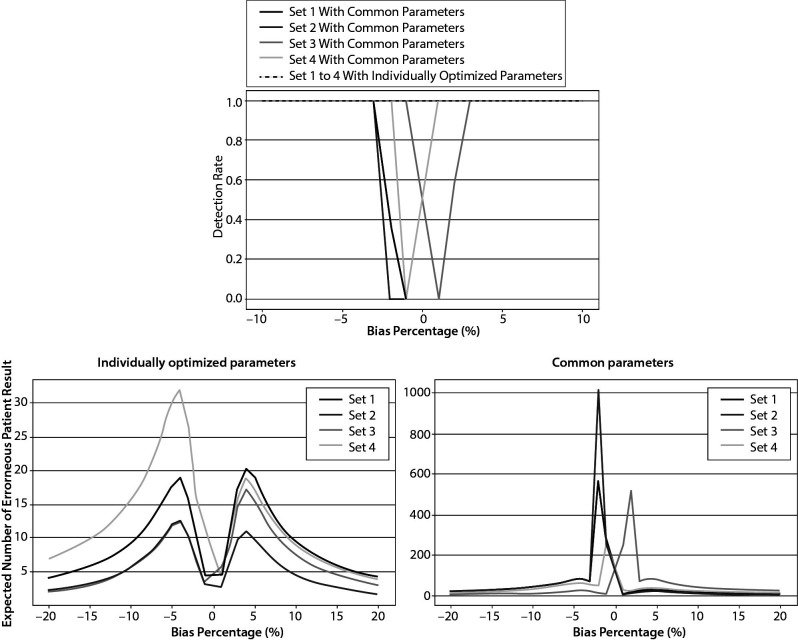
Top Panel: Comparison of bias detection rate with individually optimized and applied moving median parameters. A higher bias detection rate indicates better probability of detecting a bias. The bias detection rates for the 4 datasets with individually applied parameters have the same value of 1 and are collapsed into a single curve for clarity. Two Bottom graphs: Expected number of erroneous patient results E(N_uf_) with individually optimized and applied parameters and common parameters. A higher E(N_uf_) indicates more patient result exceeding the total allowable error as a consequence of the simulated bias. E(N_uf_) – expected number of erroneous patient results.

The E(N_uf_) for combined patient results is also highly asymmetrical across positive and negative biases (Figure 3). For example, instruments 1 and 2 with population means (140 mmol/L) nearer to the upper common control limit (140.8 mmol/L) yielded high numbers of unacceptable final patient results (E(N_uf_)) prior to bias detection of > 500 and > 100, respectively, when a small (- 2%) negative bias is introduced. The opposite is true for instrument 3 with a population mean (137 mmol/L) closer to the combined lower control limit (136.1 mmol/L) and has an E(N_uf_) of < 20 for the same - 2% bias. The bias detection rates for individually optimized parameters on the 4 datasets is 100% for all magnitudes of bias introduced (Figure 3).

## Discussion

This study compared the treatment of patient data stream from different instrument / analytical components as a single source or individually. When the PBRTQC algorithm was individually optimized and applied to the data of the individual simulated instruments, it detected bias faster and more consistently with the individual instruments showing comparable performance.

This finding is the opposite of traditional internal quality control (IQC), where the use of a common mean and control limit is associated with lower overall patient impact (*11*). This apparent contradiction is related to the fundamental difference between the two quality control practices. In IQC, the performance of the instrument is assessed by repeat measurement of a fixed material (*e.g.*, commercial QC material) over time and compared against a control limit that is determined using the analytical imprecision of the instrument. Because the same material is used across the different analysers, a reference IQC target and control limits that take into account the analytical characteristics (*i.e.*, the imprecision, which determines the control limit) of the different instruments can be determined and applied. A fixed control limit has the effect of constraining the risk of erroneous patient results for poorer performing instrument, although at the expense of higher false rejection rates. On the other hand, it relaxes the false rejection rates of better performing instruments. These have the effect of equalizing the risk of error across instruments (*11*). Nonetheless, the lack of prior comparable studies examining the effect of applying the PBRTQC algorithm on individual versus combined data sets limits the generalisability of our findings.

The optimized parameters for PBRTQC are highly dependent on the underlying patient population from which the measurements are made. It is not possible to apply a set of reference parameters across different instruments without prior optimization using the data from all instruments as a whole. When a reference parameter is applied across the different instruments, it will likely lead to a widening of control limits, thereby reducing the effectiveness of the algorithm in detecting errors compared to when the parameters were set individually for each instrument.

An important assumption of this simulation study is that the four instruments produced the patient results at the same rate. In reality, this may not necessarily hold true. An instrument producing less patient results will take a longer time to detect an error.

While combining data from different instruments can increase the data stream and hence, increase the speed of error detection, it may widen the control limits and compromising the probability of error detection. The presence of multiple instruments in the data stream may dilute the effect of the error when it only affects the select instrument, such as with reagent lot or calibration lot change on one instrument but not others.
